# Different Types of Cell Cycle- and Apoptosis-Related Gene Expressions Alter in Corticosteroid-, Vincristine-, and Melphalan-Resistant U-266 Multiple Myeloma Cell Lines

**DOI:** 10.4274/tjh.2013.0231

**Published:** 2014-09-05

**Authors:** Pelin Mutlu, Ali Uğur Ural, Ufuk Gündüz

**Affiliations:** 1 Middle East Technical University, Central Laboratory, Molecular Biology and Biotechnology R&D Center, Ankara, Turkey; 2 Bayındır Hospital, Clinic of Hematology, Ankara, Turkey; 3 Middle East Technical University, Department of Biological Sciences, Ankara, Turkey

**Keywords:** Drug resistance, Multiple myeloma, Cell Cycle, apoptosis

## Abstract

**Objective:** Deregulation of the cell cycle and apoptosis mechanisms in normal cells causes many problems, including cancer. In this study, a genome-wide expression analysis of cell cycle- and apoptosis-related genes in corticosteroid-, vincristine-, and melphalan-resistant U-266 multiple myeloma cell lines was conducted.

**Materials and Methods:** Resistant U-266 sublines were induced by application of each drug by stepwise dose increments. Resistance gained by the cells was confirmed with XTT cytotoxicity assay and microarray analyses were carried out. Among the cell cycle- and apoptosis-related gene expressions, alterations of more than 2-fold were considered significant.

**Results:** Cyclin E2 was drastically overexpressed in the vincristine-resistant subline and a general upregulation was observed for various cyclin-dependent kinases. Some of the cyclin-dependent kinase inhibitor encoding genes were downregulated in resistant sublines in general. Tumor necrosis factor receptor genes were generally downregulated in corticosteroid- and melphalan-resistant U-266 sublines. Different types of effector caspases were downregulated in all resistant sublines. Ceramide metabolism genes seemed to be changed in favor of survival, especially in the melphalan-resistant subline.

**Conclusion:** This report shows that different types of chemotherapeutic drugs alter different apoptotic and cell cycle-related gene expressions and, as a result, may cause drug-resistant phenotypes in U-266 multiple myeloma cell lines. Among those gene expressions, the most drastic increase in cyclin E2 could be important for the survival of vincristine-resistant U-266 cell lines, whereas alteration of ceramide metabolism genes could be important in melphalan resistance.

## OZET

**Amaç:** Normal hücrelerde hücre döngüsü ve apoptoz mekanizmalarının düzensiz çalışması kanser de dahil olmak üzere pek çok soruna neden olmaktadır. Bu çalışmada; kortikosteroid, vinkristin ve melfalan’a dirençli U-266 multipl miyelom hücre hatlarında hücre döngüsü ve apoptoz ile ilgili genlerin ifade düzeylerindeki farklılıklar incelenmiştir.

**Gereç ve Yöntemler:** İlaç dirençli U-266 hücre hatları her bir ilacın artan dozlarda U-266 hücrelerine uygulanması ile geliştirilmiştir. Dirençlilik gelişimi XTT sitotoksisite testleri ile gösterilmiş ve mikroarray analizi gerçekleştirilmiştir. Hücre döngüsü ve apoptoz ile ilgili olan gen ifadelerinden iki katın üzerinde olan değişiklikler anlamlı olarak kabul edilmiştir. 

**Bulgular:** Vinkristin dirençli U-266 hücre hattında siklin E2 gen ifadesinin büyük ölçüde arttığı ve çeşitli siklin bağımlı kinaz genlerinin ifadelerinde genel olarak artış olduğu gözlenmiştir. Dirençli hatlarda, bazı siklin bağımlı kinaz inhibitörü kodlayan gen ifadelerinde azalma saptanmıştır. Tümör nekroz faktörü reseptör genlerinin ifadeleri kortikosteroid ve melfalan dirençli U-266 hücre hatlarında genellikle azalmıştır. Tüm dirençli hücrelerde farklı tiplerdeki efektör kaspaz gen ifadelerinde azalma gözlenmiştir. Seramid metabolizması gen ifadelerinde ise melfalan dirençli U-266 hücrelerinin hayatta kalmalarını sağlayacak şekilde değişimler saptanmıştır. 

**Sonuç:** Bu sonuçlar, farklı kemoterapötik ilaçların farklı apoptoz ve hücre döngüsü ile ilgili gen ifadelerini değiştirerek U-266 multipl myeloma hücre hatlarında dirençliliğe neden olabileceğini göstermektedir. Bu gen ifadeleri arasında, siklin E2’deki yüksek artış vinkristine dirençli U-266 hücrelerinin hayatta kalımı için önemli olabilecekken, seramid metabolizması ile ilgili gen ifade değişiklikleri melfalan direnci açısından önemli olabileceği düşünülmektedir.

## INTRODUCTION

The emergence of drug resistance in tumor cells is a major complication for successful anticancer chemotherapy [1]. The balance between cell proliferation and apoptosis is a critical phenomenon for both development and normal tissue homeostasis. Deregulation of these processes in a normal cell results in many diseases, including cancer. Identification of genes that control cell death and apoptosis shows a linkage between apoptosis and cell cycle control mechanisms [2]. In one study it was shown that doxorubicin-resistant lung carcinoma cells exhibit altered cell cycle responses [3].

The cell cycle governs the fate of the cell [4]. Progression is controlled by external and internal signals. Cell cycle checkpoints control events throughout the cell cycle by the help of cyclins and cyclin-dependent kinases (CDKs) [4,5]. Cyclins are a family of proteins that control the progression of cells through the cell cycle by activating CDK enzymes [6,7]. At least 9 structurally related CDKs (CDK1-CDK9) have been identified. A considerable number of cyclins have also been identified to date (cyclin A-cyclin T) [4].

Cell cycle-mediated drug resistance is an important problem that must be overcome in cancer chemotherapy. It is best described as a relative insensitivity to a chemotherapeutic agent because of the position of the cells in the cell cycle. It was demonstrated when flavopiridol exposure was followed by paclitaxel in human gastric and breast cancer cells. The multiple cell cycle effects of flavopiridol, including the inhibition of different CDK activities at the G1 and G2 phases, create cell cycle arrest, which prevents cells from entering the M phase [8,9].

The process of apoptosis is controlled by a diverse range of cell signals, either extracellularly (toxins, hormones, growth factors) or intracellularly (glucocorticoids, heat, radiation, viral infection, nutrient deprivation) [10,11]. There are 2 main methods of regulation for apoptosis, either targeting mitochondria functionality or directly transducing the signal via adaptor proteins to the apoptotic mechanisms. In the mitotic process, mitochondrial proteins known as small mitochondria-derived activators of caspases are released into the cytosol following an increase in permeability and then bind to inhibitor of apoptosis proteins (IAPs), preventing the IAPs from arresting the apoptosis [12]. For the direct transduction process, 2 theories have been suggested: the tumor necrosis factor-induced (TNF) model and the Fas-Fas ligand-mediated model, both involving receptors (TNFRs) coupled to extrinsic signals [13]. There is a balance between proapoptotic (bax, bid, bak, bad) and antiapoptotic (bcl-xl, bcl-2) proteins following TNF-R1 and Fas activation [14]. Caspases, which play a central role in apoptosis, are highly conserved proteases that degrade a host of intracellular proteins to carry out the cell death program.

In addition to changes in the expression levels of particular proteins that are related to cell cycle and apoptosis, multidrug-resistant (MDR) cells exhibit major alterations in their sphingolipid composition. Sphingolipids, which include ceramides and sphingosine, are essential structural components of cell membranes that also have messenger functions that regulate the proliferation, survival, and death of cells [15,16]. Ceramide accumulation inside the cell triggers apoptosis.

A 2- to 3-fold overexpression of glucosylceramide appears to be a rather general aspect of P-glycoprotein-expressing MDR cells. An increased turnover of ceramide to glucosylceramide may allow MDR cells to escape apoptosis, since ceramide plays a major role in the regulation of apoptosis [1]. Glucosylceramide synthase overexpression has been shown to enhance resistance to doxorubicin, suggesting that inhibition of ceramide metabolism or catabolism might enhance cancer chemotherapy [15].

This study demonstrates a genome-wide expression analysis of cell cycle, apoptosis, and ceramide metabolism genes in corticosteroid (U-266/Pred)-, vincristine (U-266/Vinc)-, and melphalan (U-266/Melp)-resistant multiple myeloma cells.

## MATERIALS AND METHODS

**Cell Lines**

The human U-266 multiple myeloma cell line was obtained from Gülhane Military Medical School, Ankara, Turkey. The cells were grown in RPMI 1640 medium supplemented with 10% heat-inactivated fetal bovine serum, 1% L-glutamine, and 1% gentamicin (Biological Industries, Beit-Haemek, Israel) and were maintained at 37 °C in a humidified air atmosphere with 5% CO2. Corticosteroid (methyl prednisolone) (U-266/Pred)-, vincristine (U-266/Vinc)-, and melphalan (U-266/Melp)-resistant sublines were developed from the original U-266 cells by applying each drug separately in dose increments. The XTT cell viability assay was performed for original and drug-resistant sublines with the Cell Proliferation Assay Kit (Biological Industries). The fold of resistance values of the sublines were previously reported [17].

**RNA Isolation, cDNA Synthesis, and Target Preparation**

RNA isolation from all cells was performed using TRI reagent (Sigma, St. Louis, MO, USA) according to the manufacturer’s instructions. All RNA samples were prepared as duplicates for statistical analysis. RNA concentrations were adjusted to at least 2.5 µg/µL. cRNA synthesis, target hybridization, and scanning were performed at the Ankara University Biotechnology Institute (Ankara, Turkey). cDNAs were synthesized from total RNA by One-Cycle Target Labeling Assay (Affymetrix, Santa Clara, CA, USA) according to the manufacturer’s instructions. Second-strand cDNA synthesis, biotin-labeled cRNA synthesis, and cRNA fragmentation were performed using the Affymetrix GeneChip Kit. Hybridization was conducted at 45 °C and 60 rpm for 16 h in an Affymetrix Gene Chip Hybridization Oven 640 and the arrays were stained using a hybridization stain kit according to the instructions in the technical manual. Washing and staining were performed in an Affymetrix GeneChip Fluidics Station 450 with EukGE-WS2v5 fluidics script. The arrays were scanned in an Affymetrix GeneChip Scanner 3000.

**Data Analysis**

Affymetrix GeneChip Operating Software and GeneSpring GX 7.3.1 Software (Agilent Technologies, Inc., Santa Clara, CA, USA) were used for expression data analysis. The data were normalized by robust multichip analysis method. The gene expression levels were calculated at the level of oligonucleotide sets by using the median polish method [18] and log 2 equivalents were calculated. Statistically significant data were selected by independent sample t-test (p<0.05) between duplicate data for resistant and original cells. The genes upregulated and downregulated more than 2-fold were considered and, by using the Kyoto Encyclopedia of Genes and Genomes pathway, grouping of data that could contribute to drug resistance was performed. Finally, the genes that encode proteins related to cell cycle and apoptosis were selected and evaluated for possible relations to drug resistance phenotype. The microarray data of this study were confirmed with the RT-PCR results of the representative genes (MDR1, MRP1, Bcl-2, LRP, and BCRP) of the MDR phenotype (unpublished data).

## RESULTS

Alterations in cell cycle- and apoptosis-related genes in drug-resistant U-266 multiple myeloma sublines are shown in Table 1. Different types of cell cycle-encoding genes and CDKs, especially CDK6 (-119-fold) and cyclin D3 (-104-fold), were downregulated, whereas CDK inhibitor encoding genes (CDKN2B and CDKN2A) were upregulated in the corticosteroid-resistant U-266 cell line. However, cyclin E2 and the 2 recently identified subunits of E2F, E2F7 and E2F8, were drastically overexpressed in vincristine resistance. In melphalan resistance, some of the CDK inhibitor encoding genes (CDKN1A and CDKN1C) were downregulated.

TNF alpha-induced protein 3 (TNFAIP3) and TNF receptor superfamily member 10d (TNFRSF10) were downregulated in the U-266/Pred subline. However, TNF-inducible gene 6 protein (TNFAIP6) and TNF alpha-induced protein 8 (TNFAIP8) were upregulated in vincristine resistance.

Caspase-3, -6, and -7 are called effector caspases, which are downstream caspases that in turn cleave other protein substrates within the cell to trigger the apoptotic processes. A general downregulation of different types of caspases was observed in drug-resistant sublines (Table 1).

The baculoviral IAP repeat-containing (BIRC) group of genes, which belongs to a family that inhibits apoptosis, was overexpressed in corticosteroid and vincristine resistance but not in the melphalan-resistant subline. The Bcl-related genes (BNIP1 and BNIP3) were upregulated in the U-266/Vinc subline whereas they were downregulated in U-266/Pred. The most common Bcl-2 and Bcl-XL genes of this family were not significantly altered in these sublines, as confirmed by RT-PCR and western blot analysis (data not shown). The programmed cell death-related genes (PDCD11 and PDCD4) were also upregulated in the vincristine-resistant subline and downregulated in the corticosteroid-resistant one, which is parallel to Bcl-related gene expressions.

Table 1 also shows the altered gene expressions in ceramide metabolism. The LAG1 homolog ceramide synthase 6 (LASS 6), sphingomyelin phosphodiesterase (SMPD3), and degenerative spermatocyte homolog 1 lipid desaturase (DEGS1) genes were upregulated while UDP-glucose ceramide glycosyltransferase (UGCG) and N-acylsphingosine amidohydrolase (ASAH1) genes were downregulated in the corticosteroid-resistant subline; all of these regulations cause accumulation of ceramide inside the cells. However, the LASS 6 and SMPD1 genes were downregulated in melphalan resistance, leading to degradation to ceramide inside the cells. 

## DISCUSSION

The cyclin E2 gene was 487-fold overexpressed in the U-266/Vinc subline. Cyclin E is a member of the cyclin family, which is required for the transition from the G1 to the S phase [19]. On the other hand, 2 of the transcription factor E2F subunits, E2F7 and E2F8, were highly overexpressed in the vincristine-resistant U-266 subline. E2F7 and E2F8 have an important role in DNA damage response. They repress E2F site-dependent transcription in a pRb-independent manner and delay cell cycle progression. High levels of E2F7 and E2F8 expression were shown as intrinsically lower sensitivity to E2F1-dependent apoptosis [20]. In order to overcome the mitotic inhibition of vincristine, the cells seem to upregulate the expression of their cell cycle-related genes. The breast cancer type 2 susceptibility (BRCA2) and CDKN1A interacting protein is also known as BCCIP. This gene product was isolated on the basis of its interaction with BRCA2 and p21 proteins. Functional studies indicate that this protein may be an important cofactor for BRCA2 in tumor suppression and a modulator of CDK2 kinase activity via p21 [21]. p21 is an important tumor suppressor protein that is regulated by p53. BCCIP gene expression was downregulated in both the corticosteroid- and vincristine-resistant U-266 sublines. Therefore, this downregulation may have a significant effect on cell cycle stimulation. In melphalan resistance, CDK inhibitor encoding genes (CDKN1A and CDKN1C) were downregulated, which may again be correlated with cell proliferation.

TNF acts via the TNF receptor (TNF-R) and is part of the extrinsic pathway for triggering apoptosis [13]. TNFRSF10, which is a proapoptotic gene, was shown to be upregulated in melanoma cells destined to undergo apoptosis [22]. In this study, this gene was downregulated in the corticosteroid-resistant U-266 subline, indicating a change towards cell survival. TNFAIP8 and TNFAIP6 (TSG-6) genes were both upregulated in vincristine resistance. TNFAIP8 is known as an antiapoptotic protein, and TSG-6 is involved in the context of inflammation and is often associated with extracellular matrix remodeling [23]. TSG-6 is a secretory protein that has been identified as a member of the hyaluronate binding protein family [24]. It binds extracellular matrix glycosaminoglycan hyaluronan [25]. It was also reported that TSG-6 modulates the interaction of hyaluronan with CD44 marker and thus can interfere with CD44-mediated interactions of lymphoid cells with hyaluronan in postcapillary venules [26]. CD44 is involved in various cell adhesion events, including lymphocyte migration, hematopoiesis, and tumor metastasis [27]. TNFRSF19, which activates the JNK signaling pathway and is capable of inducing apoptosis by a caspase-independent mechanism [28], was downregulated in the U-266/Melp subline. From these results, it can be seen that resistant sublines tend to escape apoptosis by regulating different types of TNF-related genes. Although different drugs trigger different regulatory pathways, they all tend to maintain the survival of the cell. 

Different types of the effector caspases that trigger the apoptotic processes were downregulated in all resistant U-266 sublines. BIRC-related genes were overexpressed in corticosteroid- and vincristine-resistant sublines. The BIRC group of genes inhibits apoptosis by binding to TNF receptor-associated factors TRAF-1 and TRAF-2 [29]. BAG and BNIP are Bcl-related genes. These genes were upregulated in the vincristine-resistant U-266 subline and downregulated in the corticosteroid-resistant one. BAG is a Bcl-2-associated multifunctional prosurvival molecule that binds to Hsp70/Hsc70 proteins. BNIP proteins, on the other hand, have roles in apoptosis in regulating the expression of genes associated with cell apoptosis, growth inhibition, and cell proliferation [30]. Programmed cell death is death of a cell mediated by an intracellular program. The PDCD11, PDCD4, and PDCD2 genes were overexpressed in the U-266/Vinc subline but downregulated in corticosteroid resistance, as in the case of Bcl-related genes. PDCD11 is an NFκB binding protein that colocalizes in the nucleus [31]. The PDCD4 gene encodes a protein localized to the nucleus in proliferating cells. It has recently been demonstrated to be a new tumor suppressor gene involved in colon carcinogenesis [32]. Its role in resistance of multiple myeloma cells is not clear. The PDCD2 gene encodes a nuclear protein expressed in a variety of tissues. Expression of this gene has been shown to be repressed by Bcl-6, suggesting that Bcl-6 regulates apoptosis by its effects on PDCD2 [33].

Ceramides are a family of lipid molecules. The most well-known functions of ceramides as cellular signals include regulating the differentiation, proliferation, programmed cell death, and apoptosis of cells [34]. Increased intercellular ceramide levels are associated with apoptosis and so downregulation of genes that are responsible for ceramide synthesis or upregulation of genes that have roles in ceramide clearance is effective in cell proliferation. Constitutive degradation of sphingolipids and glycosphingolipids takes place in the acidic subcellular compartments, the late endosomes and the lysosomes. Ceramide can be further hydrolyzed by acid ceramidase to form sphingosine and a free fatty acid, both of which are able to leave the lysosome, in contrast to ceramide. In this study, LASS 6 and SMPD1 genes were downregulated in melphalan resistance, leading to degradation to ceramide inside the cells. On the other hand, the LASS 6, SMPD3, and DEGS1 genes were upregulated while UGCG and ASAH1 were downregulated in the corticosteroid-resistant subline. These regulations cause accumulation of ceramide inside corticosteroid-resistant cells. The upregulation of ceramidase, sphingosine kinase, and glucosylceramide synthase and the downregulation of ceramide synthase genes can be important for drug resistance phenotypes since they lower the ceramide levels within the cell. In the literature, there are some studies that support this hypothesis [15]. Glucosylceramide synthase overexpression has been shown to enhance resistance to doxorubicin and alterations of ceramide/sphingosine 1-phosphate were shown to be involved in the regulation of resistance to imatinib in the K562 chronic myeloid leukemia cell line [35]. Several anticancer agents, including the cytotoxic retinoid fenretinide (4-HPR), have been shown to act by increasing tumor cell ceramide via de novo synthesis [15]. Expression of glucosylceramide synthase mRNA in the vincristine-resistant KBV200 cell line was shown in association with multidrug resistance [36].

In conclusion, in the vincristine-resistant U-266 multiple myeloma cell line, cyclin E2 gene expression was drastically increased, whereas ceramide metabolism genes were altered only in melphalan resistance in favor of survival in the U-266 cell line. However, TNF receptor genes were generally downregulated in corticosteroid- and melphalan-resistant U-266 sublines. This shows that different types of chemotherapeutic drugs alter different apoptotic and cell cycle-related gene expressions. On the other hand, all of the anticancer agents studied here are currently used in the clinical treatment of multiple myeloma. In vitro, different drug-resistant U-266 multiple myeloma cell lines show altered patterns of gene expressions related to apoptosis and the cell cycle. From a clinical perspective, this in vitro study may be a guide to clinicians for the development of new treatment strategies in drug-resistant cases. In the clinical setting, patients give different responses to the same antimyeloma regimens. By figuring out the patient profile, like in the case of our in vitro study, different types of drug combinations can be used in order to overcome the drug resistance phenotype. However, the results of this study only provide preliminary insight into this phenomenon.

**Acknowledgments **

We gratefully acknowledge the Ankara University Biotechnology Research Center for technical assistance. This study was supported by TÜBİTAK (SBAG 3297), Turkey.

**Conflict of Interest Statement**

The authors of this paper have no conflicts of interest, including specific financial interests, relationships, and/ or affiliations relevant to the subject matter or materials included.

## Figures and Tables

**Table 1:**
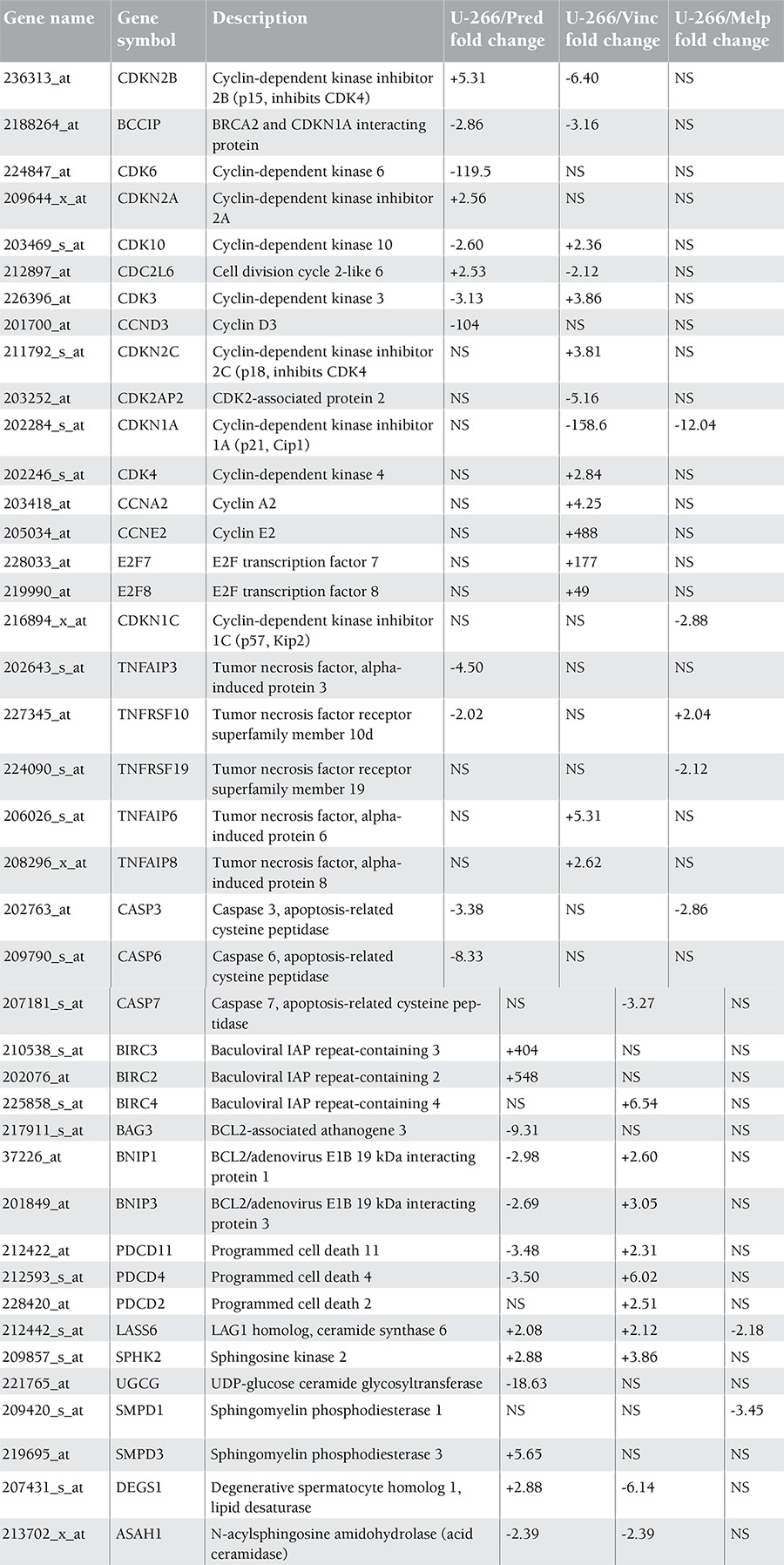
Expression levels of cell cycle- and apoptosis-related genes in corticosteroid-, vincristine-, and melphalan-resistant U-266 cell lines.
